# Association between amide proton transfer-weighted imaging biomarkers and Gleason score in prostate cancer: a study on differential diagnosis from benign prostatic hyperplasia

**DOI:** 10.3389/fonc.2026.1780850

**Published:** 2026-03-20

**Authors:** Jie Feng, Jun Wang, Jianliang Wang, Xuelian Chen, Mengqin Xu, Hongyan Wang

**Affiliations:** Department of Radiology, Kunshan First People’s Hospital, Affiliated Kunshan Hospital of Jiangsu University, Suzhou, China

**Keywords:** amide proton transfer imaging, Gleason score, PI-RADS v2.1, prostate cancer, PSA gray zone, risk stratification

## Abstract

**Background and purpose:**

The non-invasive differentiation of prostate cancer (PCa) from benign prostatic hyperplasia (BPH) and the accurate assessment of tumor aggressiveness remain critical clinical challenges. This study aimed to evaluate the diagnostic performance of amide proton transfer-weighted (APTw) MRI and, specifically, to determine its incremental value over the standard Prostate Imaging–Reporting and Data System (PI-RADS v2.1) and serum PSA.

**Methods:**

In this retrospective study, 160 patients (86 PCa, 74 BPH) underwent 3.0T multi-parametric MRI, including APTw imaging. Quantitative APTw values were compared with Apparent Diffusion Coefficient (ADC) values and PI-RADS v2.1 scores. Receiver operating characteristic (ROC) analysis was employed to evaluate the diagnostic performance of individual parameters and combined models. Subgroup analyses were performed for lesions in the Transition Zone (TZ) and patients in the PSA “gray zone” (4–10 ng/mL). Additionally, the correlation between APTw values and the Gleason Score (GS) was assessed.

**Results:**

APTw values were significantly higher in the PCa group compared to the BPH group (2.1% ± 0.5% vs. 1.3% ± 0.6%, p < 0.001). While PI-RADS v2.1 alone showed high diagnostic efficacy (AUC = 0.875), the combined model (APTw + PI-RADS v2.1) achieved the highest accuracy with an AUC of 0.915, significantly outperforming PI-RADS alone (p < 0.05). In the challenging PSA “gray zone”, APTw imaging maintained robust diagnostic performance (AUC = 0.80), significantly outperforming serum PSA (AUC = 0.75). Furthermore, a significant positive correlation was observed between APTw values and GS (Spearman’s ρ = 0.407, p < 0.001), and APTw successfully differentiated high-risk (GS ≥ 8) from low- and intermediate-risk PCa (AUC = 0.715).

**Conclusion:**

APTw imaging correlates with tumor aggressiveness and provides excellent diagnostic performance for differentiating PCa from BPH. Crucially, it offers incremental diagnostic value to the standard PI-RADS v2.1 assessment and demonstrates robust utility in challenging clinical scenarios, such as the PSA gray zone, thereby serving as a valuable non-invasive biomarker for risk stratification.

## 1 Introduction

Prostate cancer (PCa) is the fourth most frequently diagnosed malignancy globally and ranks as a leading cause of cancer-related morbidity in the male genitourinary system (1). In contrast, benign prostatic hyperplasia (BPH) is a highly prevalent non-malignant condition affecting the aging male population. The clinical management of PCa is critically dependent on tumor aggressiveness. Timely interventions, such as surgery, radiotherapy, or chemotherapy, are essential for high-risk patients to optimize outcomes (2). The Gleason Score (GS) remains the cornerstone histopathological system for assessing PCa aggressiveness, effectively reflecting tumor differentiation and heterogeneity (3–5).

Magnetic Resonance Imaging (MRI) has revolutionized the non-invasive management of prostate diseases. Multi-parametric MRI (mpMRI), encompassing anatomical T2-weighted imaging and functional diffusion-weighted imaging (DWI), is currently the primary modality for tumor detection, localization, and local staging (6). Furthermore, imaging features derived from standard MRI, alongside advanced radiomic analysis and machine learning models, have been increasingly utilized to non-invasively predict tumor aggressiveness and correlate with the Gleason Score, thereby providing crucial guidance for biopsy decisions and risk stratification (7). While the current standard Prostate Imaging–Reporting and Data System (PI-RADS v2.1) provides standardized assessment, it relies heavily on anatomical and diffusion-weighted imaging, which can be limited by subjective interpretation and overlap in imaging features between PCa and stromal BPH, particularly in the transition zone (8). Therefore, advanced functional imaging techniques providing molecular information are needed to improve diagnostic specificity.

In recent years, Amide Proton Transfer-weighted (APTw) imaging has emerged as a novel molecular MRI technique based on the Chemical Exchange Saturation Transfer (CEST) mechanism. By selectively saturating amide protons in mobile cellular proteins and peptides, APTw imaging detects the chemical exchange between these protons and bulk water protons, generating a contrast that reflects tissue proteomic composition (9–11). This technique has shown promising applications in oncology, particularly for diagnosing central nervous system tumors, where malignant proliferation is linked to altered protein synthesis (12).

The biological rationale for applying APTw imaging to prostate cancer lies in its ability to quantify intracellular protein content, which serves as a potential surrogate for tumor aggressiveness. Theoretically, the aggressiveness of prostate cancer, as reflected by the Gleason Score, is intrinsically linked to cellular density and protein synthesis rates. High-grade tumors exhibit rapid proliferation and hypercellularity, leading to an upregulation of protein synthesis and an increased concentration of mobile cytosolic proteins (13, 14). Consequently, high-grade tumors are expected to exhibit elevated APTw signals compared to low-grade tumors or benign tissues. This pathophysiological link suggests that APTw imaging could serve as a functional biomarker for the non-invasive prediction of the Gleason Score.

Currently, the definitive assessment of GS relies on invasive procedures. Transrectal or transperineal biopsies carry risks of bleeding, infection, and sampling error, potentially underestimating disease severity (15–17). Although radical prostatectomy provides a complete specimen, it is associated with significant costs and surgical risks (18, 19). While serum prostate-specific antigen (PSA) is the standard screening tool, its limitations, including high false-positive rates and poor specificity in the diagnostic “gray zone” (4–10 ng/mL), are well-documented (20). Preliminary research suggests APTw signals in PCa are significantly higher than in benign tissues (21) and correlate with tumor grade (22). However, the added value of APTw over the current clinical standard (PI-RADS v2.1) and its performance in challenging diagnostic subgroups (e.g., transition zone lesions and PSA gray zone) remain to be fully quantified.

Therefore, this study aims to elucidate the clinical utility of APTw imaging by evaluating its diagnostic value in differentiating PCa from BPH and its correlation with Gleason scores for risk stratification. Specifically, we sought to determine whether incorporating APTw imaging provides incremental diagnostic value over standard PI-RADS v2.1 assessment and serum PSA, particularly in clinically challenging scenarios.

## 2 Materials and methods

### 2.1 Clinical data and patient selection

This retrospective study was approved by the Ethics Committee of Kunshan First People’s Hospital, and the requirement for informed consent was waived due to the retrospective nature of the study. We screened patients who underwent prostate MRI, including an APT sequence, at our institution between January 2022 and June 2024. The inclusion criteria were: (1) complete MRI imaging data with diagnostic-quality APT sequences; (2) no prior treatment for prostate disease (e.g., surgery, hormone therapy, radiotherapy) before MRI; (3) availability of a pathological Gleason Score (GS) obtained via ultrasound-guided systematic biopsy or radical prostatectomy within two weeks after MRI; and (4) availability of serum Prostate-Specific Antigen (PSA) test results within two weeks before MRI. The exclusion criteria were: (1) severe motion or susceptibility artifacts on MRI images affecting ROI placement; (2) any prior treatment for prostate cancer; (3) history or imaging findings suggestive of other synchronous malignancies.

### 2.2 MRI acquisition protocol

All examinations were performed using a 3.0T MRI scanner (Philips Ingenia) with a 16-channel phased-array surface coil. The scanning center was positioned 2 cm above the pubic symphysis. To strictly control image quality and minimize artifacts, an abdominal belt was applied to reduce respiratory motion. Patients were instructed to fast for 4 hours and evacuate their bowels to reduce intestinal peristalsis and to maintain a moderately filled bladder before the exam. The imaging protocol included: sagittal and coronal T2-weighted spectral attenuated inversion recovery (SPAIR), axial T1-weighted turbo spin-echo (TSE), axial T2-weighted TSE, diffusion-weighted imaging (DWI with b-values of 50 and 1500 s/mm²), dynamic contrast-enhanced (DCE) imaging, and the APT-weighted sequence. The APT sequence was acquired using a 3D turbo spin-echo readout with a saturation pulse of 2 μT amplitude and 500 ms duration. Detailed sequence parameters are provided in **Supplementary Table 1**.

### 2.3 Image processing and APTw analysis

Image post-processing was performed on a Philips IntelliSpace Portal workstation. The APT images were co-registered and fused with axial T2-weighted images. To ensure data reproducibility and standardization, B0 field inhomogeneity correction was automatically performed by the workstation algorithms prior to APTw map generation. The region of interest (ROI) was carefully placed on the axial slice showing the largest cross-sectional area of the lesion, referencing T2-weighted and high-b-value DWI images. Precautions were taken to avoid areas of necrosis, hemorrhage, calcification, or cystic components. Two senior radiologists visually inspected all raw APTw images; cases with severe geometric distortion or susceptibility artifacts caused by rectal gas were excluded. The primary quantitative parameter was the amide proton transfer-weighted (APTw) signal, calculated as the magnetization transfer ratio asymmetry (MTRasym) at 3.5 ppm. The workstation automatically generated the mean (APTmean) values for each ROI, which were used for statistical analyses. Measurements were performed independently by two radiologists (Rater A and Rater B, each with >5 years of experience in genitourinary MRI), blinded to pathological results. Each radiologist measured each ROI three times, and the average was recorded. The final APTw value for each lesion was the mean of the measurements from both radiologists. The intraclass correlation coefficient (ICC) was utilized to evaluate inter-observer agreement.

### 2.4 Radiographic evaluation

To evaluate the incremental value of APTw, standard multi-parametric MRI metrics were also assessed. The two radiologists, blinded to the pathological outcomes, independently assigned a PI-RADS v2.1 score to each lesion based on T2-weighted, DWI, and DCE sequences (8). Additionally, quantitative Apparent Diffusion Coefficient (ADC) values were measured on the corresponding ADC maps using the same ROIs defined for APTw analysis. For inconsistent PI-RADS scores, a consensus was reached through discussion.

### 2.5 Pathological evaluation

Pathological diagnosis served as the reference standard. All patients underwent ultrasound-guided systematic biopsy (sampling the bilateral peripheral zone and transition zone) or radical prostatectomy. Histopathological slides were reviewed by a team of senior pathologists. According to the 2019 International Society of Urological Pathology (ISUP) consensus (23), the Gleason Score (GS) was assigned. Immunohistochemical staining (positive for AMACR/P504s and negative for P63) was used for confirmation in challenging cases. Based on the final GS, prostate cancer cases were categorized into two risk groups: a high-risk group (GS ≥ 8) and a low- and intermediate-risk group (GS < 8) (24).

### 2.6 Statistical analysis

Statistical analyses were performed using R software (version 4.2.2) (25). Continuous variables were compared using Independent samples t-tests or Mann-Whitney U tests depending on normality, and categorical variables were compared using Chi-square or Fisher’s exact tests. Receiver operating characteristic (ROC) curve analysis was employed to evaluate the diagnostic performance of APTw, ADC, PI-RADS v2.1, and PSA. The Area Under the Curve (AUC), sensitivity, and specificity were calculated. The DeLong test was used to compare the AUCs of different models (e.g., PI-RADS vs. Combined Model). Subgroup analyses were performed to evaluate diagnostic performance in specific clinical scenarios: (1) lesions located in the Transition Zone (TZ), and (2) patients with PSA levels in the diagnostic “gray zone” (4–10 ng/mL). Correlations between APTw values and Gleason Scores were assessed using Spearman’s rank correlation coefficient. Multivariate logistic regression was used to identify independent predictors, reporting odds ratios (OR) with 95% confidence intervals (CI). A two-sided p-value < 0.05 was considered statistically significant.

## 3 Results

### 3.1 Baseline characteristics of BPH and PCa patients

This retrospective analysis enrolled a total of 160 patients, including 86 with histopathologically confirmed PCa and 74 diagnosed with BPH. **Table 1** provides a detailed overview of the baseline demographic and clinical profiles of the patients included in the study. There was no significant difference in age between the BPH and PCa groups (73.8 ± 4.7 years vs. 73.3 ± 4.7 years, p=0.514). Nonetheless, serum PSA levels were notably elevated in the PCa group in comparison to the BPH group(14.7 ± 7.4 ng/mL vs. 9.2 ± 3.4 ng/mL, p<0.001).

**Table 1:** Demographic, clinical, and imaging characteristics of the study population.

Characteristic	BPH	PCa	P-value
Age (years)	73.8 ± 4.7	73.3 ± 4.7	0.517
Body mass index (kg/m^2^)	21.7 ± 1.6	21.7 ± 1.8	0.971
Height (cm)	167.7 ± 5.2	168.1 ± 5.9	0.627
Weight (kg)	61.0 ± 5.9	61.3 ± 6.1	0.752
Smoking history	48 (64.9%)	55 (64.0%)	1
Alcohol history	51 (68.9%)	59 (68.6%)	1
Hypertension	37 (50.0%)	35 (40.7%)	0.308
APTw (%)	1.3 ± 0.6	2.1 ± 0.5	<0.001
PSA (ng/mL)	9.2 ± 3.4	14.7 ± 7.4	<0.001
Prostate volume (mL)	65.3 ± 17.5	48.7 ± 9.2	<0.001
PSA density (ng/mL^2^)	0.1 ± 0.0	0.4 ± 0.1	<0.001
ADC (× 10–^3^ mm^2^/s)	1.26 ± 0.18	0.86 ± 0.15	<0.001
PI-RADS v2.1 Score			<0.001
Score 1-2	52 (70.3%)	3 (3.5%)	
Score 3	18 (24.3%)	12 (14.0%)	
Score 4-5	4 (5.4%)	71 (82.5%)	
Lesion Location			<0.001
Peripheral Zone (PZ)	11 (14.9%)	60 (69.8%)	
Transition Zone (TZ)	63 (85.1%)	26 (30.2%)	

Data are presented as mean ± standard deviation for continuous variables and n (%) for categorical variables. APTw, amide proton transfer-weighted; PSA, prostate-specific antigen; BPH, benign prostatic hyperplasia; PCa, prostate cancer; ADC, apparent diffusion coefficient; PI-RADS, Prostate Imaging–Reporting and Data System. Statistical Analysis: Independent samples t-test or Mann-Whitney U test was used for continuous variables depending on normality; the Chi-square test or Fisher’s exact test was used for categorical variables. Bold values indicate statistical significance (p < 0.05).

Regarding standard MRI metrics, the PCa group exhibited features typical of malignancy, including significantly lower mean ADC values compared to the BPH group (0.86 ± 0.15 vs. 1.26 ± 0.18 × 10–^3^ mm^2^/s, p < 0.001). The distribution of PI-RADS v2.1 scores also differed significantly: 82.5% of PCa patients were scored as PI-RADS 4–5, whereas 70.3% of BPH patients were scored as PI-RADS 1–2 (p < 0.001). Furthermore, lesion distribution varied, with PCa predominantly located in the Peripheral Zone (69.8%) and BPH mainly in the Transition Zone (85.1%). Quantitative analysis confirmed that APTw values were significantly higher in PCa lesions (2.1 ± 0.5%) than in BPH nodules (1.3 ± 0.6%, p < 0.001).

### 3.2 Inter-observer agreement for APTw measurements

**Figure 1:**
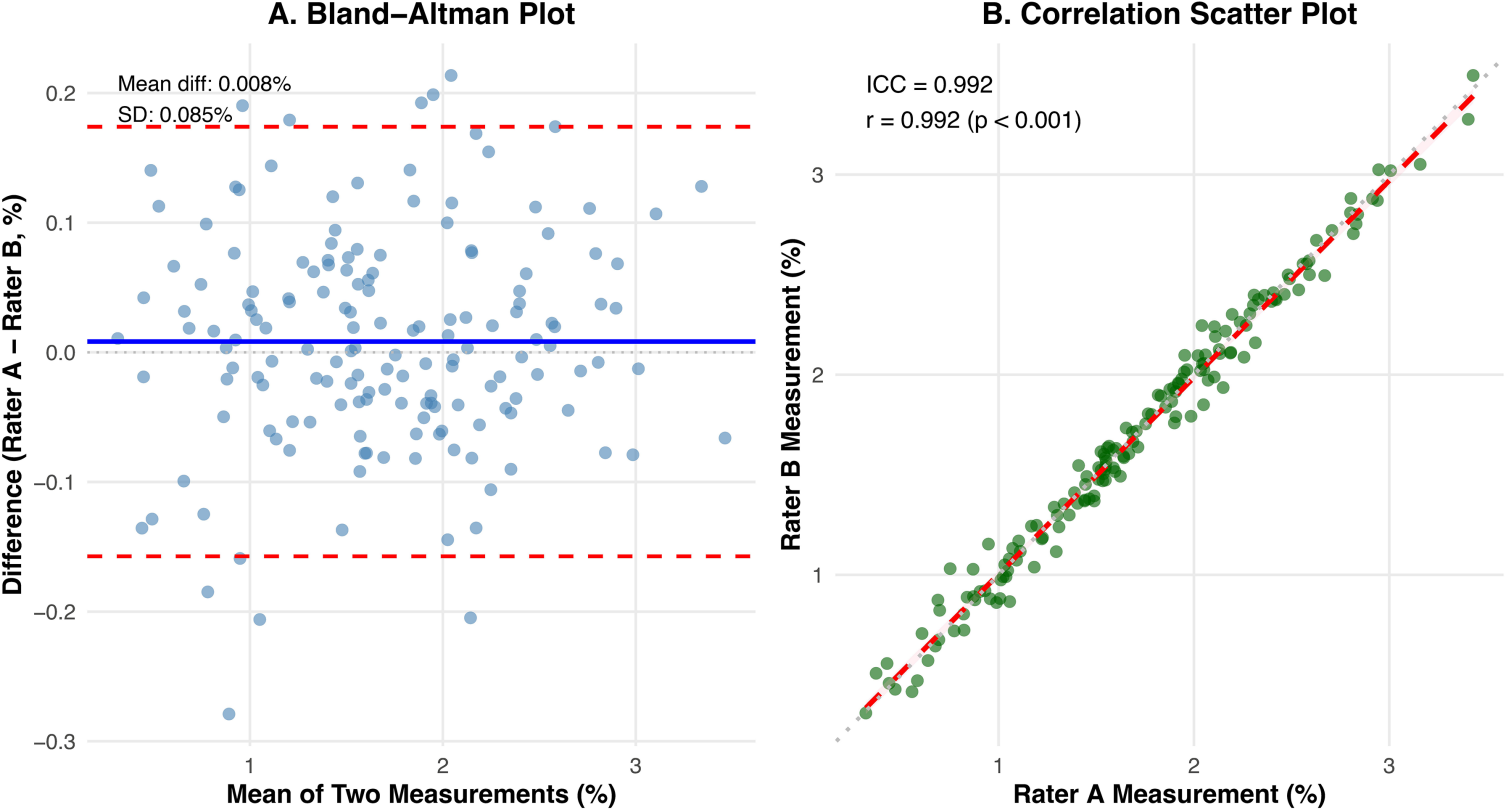
Inter-observer agreement analysis for APTw measurements. **(A)** Bland-Altman plot showing the agreement between Rater A and Rater B, with a minimal mean difference of 0.008%. **(B)** Correlation scatter plot demonstrating excellent consistency between measurements from the two radiologists (ICC = 0.992).

An excellent amount of inter-observer agreement was obtained for APTmean measurements, demonstrating an ICC of 0.992 (95% CI: 0.989-0.994). Bland-Altman analysis indicated a minimal mean inter-reader difference of 0.008%, with 95% limits of agreement between -0.159% and 0.175% (**Figure 1A**). Furthermore, measurements from the two radiologists correlated strongly (Pearson’s r = 0.992, p < 0.001; **Figure 1B**).

### 3.3 Comparative diagnostic performance: APTw vs. PI-RADS v2.1 and ADC

**Figure 2:**
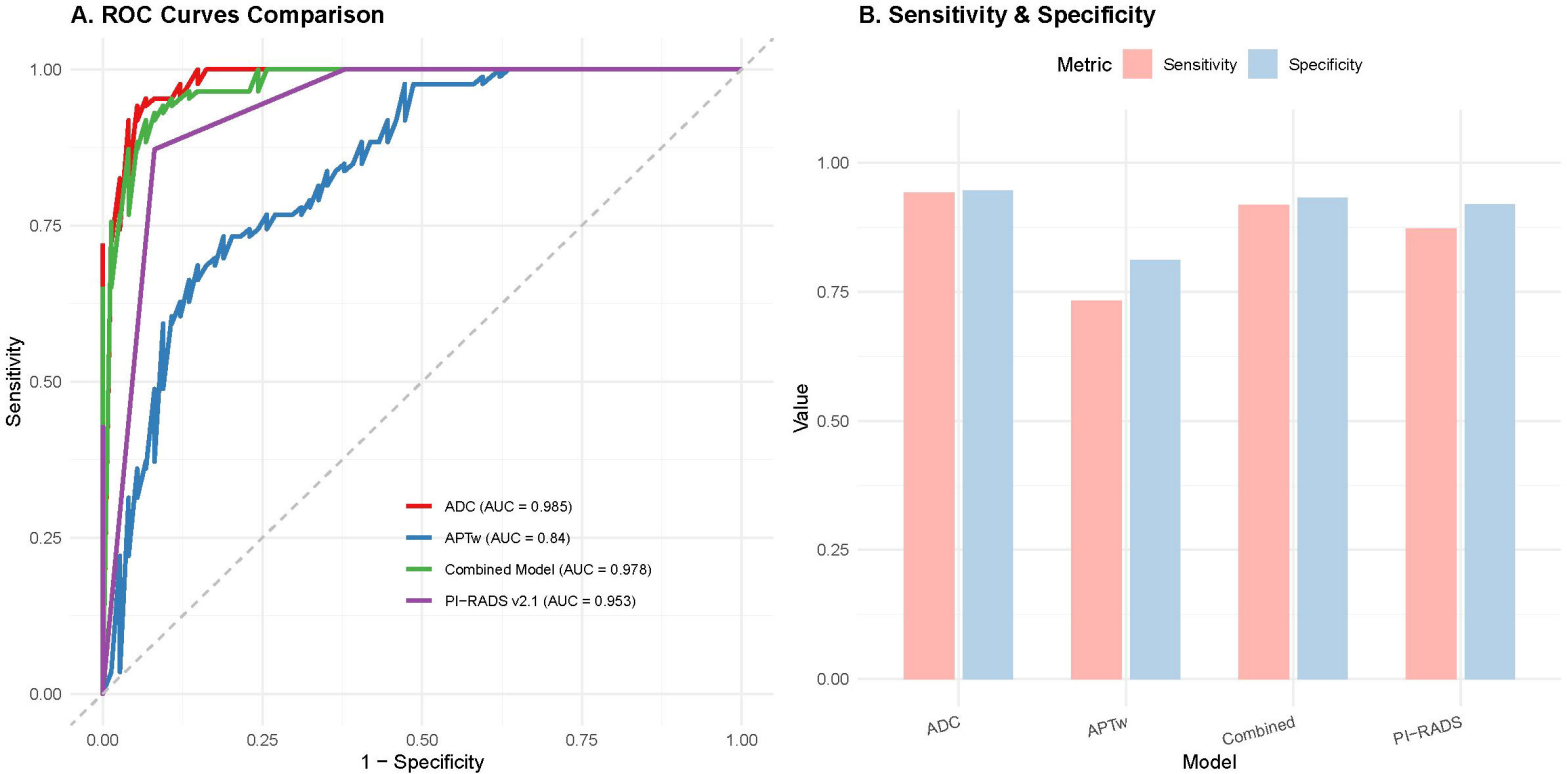
Comparative diagnostic performance of multi-parametric MRI metrics. **(A)** ROC curves distinguishing PCa from BPH using APTw, ADC, PI-RADS v2.1, and the Combined Model (APTw + PI-RADS). Note that the Combined Model (purple line) yields the highest AUC. **(B)** Bar chart comparing the sensitivity and specificity of each parameter. The combination of APTw and PI-RADS v2.1 significantly improves diagnostic accuracy compared to PI-RADS alone.

To verify the incremental value of APTw imaging, we compared its diagnostic efficacy against standard multi-parametric MRI indicators (**Table 2**, **Figure 2**). In the individual parameter analysis, PI-RADS v2.1 achieved the highest AUC of 0.875 (95% CI: 0.820–0.930), followed by ADC (AUC = 0.852) and APTw (AUC = 0.840). All three MRI-derived parameters significantly outperformed serum PSA alone (AUC = 0.736).

**Table 2:** Comparative diagnostic performance of individual parameters and combined models for differentiating PCa from BPH.

Model	AUC	95% CI	Cutoff	Sensitivity (%)	Specificity (%)	PPV (%)	NPV (%)
Individual parameters							
PSA	0.736	0.658 - 0.814	14.4	46.5	94.6	90.9	60.3
ADC	0.852	0.795 - 0.908	0.95	82.6	85.1	86.6	80.8
PI-RADS v2.1	0.875	0.820 - 0.930	>3	84.9	89.2	90.1	83.5
APTw	0.84	0.778 - 0.902	1.7	73.3	81.1	81.8	72.3
Combined models							
APTw + PSA	0.888	0.839 - 0.937	–	80.2	83.8	85.2	78.5
APTw + PI-RADS	0.915*	0.870 - 0.960	–	90.7	91.9	92.9	89.5

APTw, amide proton transfer-weighted; PSA, prostate-specific antigen; ADC, apparent diffusion coefficient; PI-RADS, Prostate Imaging–Reporting and Data System v2.1; AUC, area under the receiver operating characteristic curve; CI, confidence interval; PPV, positive predictive value; NPV, negative predictive value. Comparison: The combined model (APTw + PI-RADS) demonstrated the highest diagnostic accuracy. * p < 0.05 indicates a statistically significant difference compared to PI-RADS v2.1 alone (DeLong test), demonstrating the incremental diagnostic value of APTw imaging.

Most importantly, the combined model incorporating APTw and PI-RADS v2.1 yielded the highest diagnostic accuracy, with an AUC of 0.915 (95% CI: 0.870–0.960). This combined performance was significantly superior to PI-RADS v2.1 alone (p < 0.05), indicating that APTw provides added diagnostic value. At the optimal cutoff, the combined model achieved a sensitivity of 90.7% and a specificity of 91.9%.

### 3.4 Subgroup analysis: addressing clinical challenges

**Figure 3:**
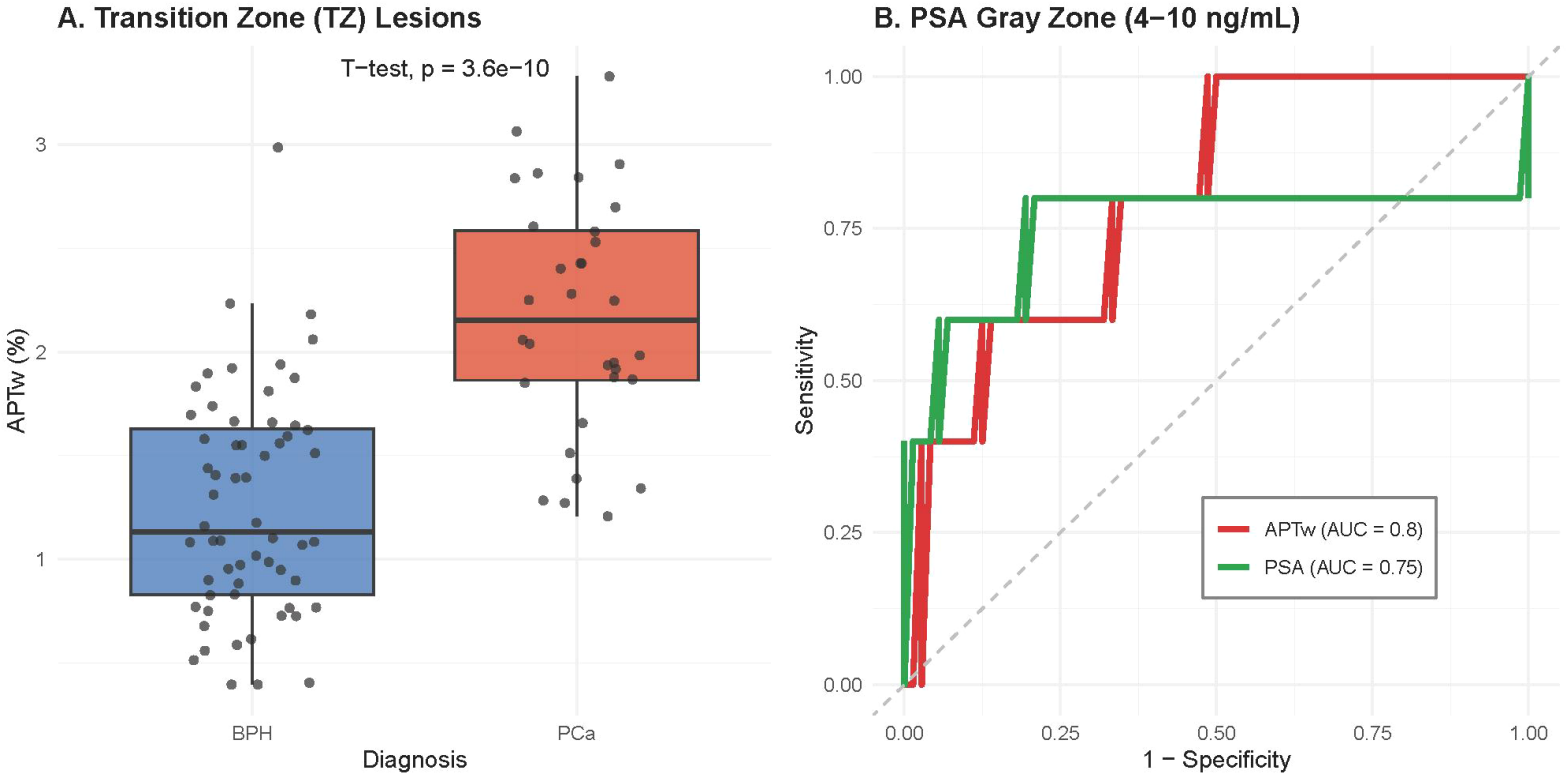
Subgroup analysis addressing clinical diagnostic challenges. **(A)** Box plot of APTw values in Transition Zone (TZ) lesions. APTw significantly differentiates TZ-PCa from BPH nodules (p < 0.001), demonstrating robustness in the central gland. **(B)** ROC curves for patients in the PSA “gray zone” (4–10 ng/mL). APTw (red line, AUC = 0.80) maintains high diagnostic accuracy in this challenging population, significantly outperforming serum PSA (green line, AUC = 0.75).

We performed targeted subgroup analyses to evaluate APTw performance in clinically difficult scenarios (**Figure 3**). Transition Zone (TZ) Lesions: Differentiating PCa from stromal BPH in the transition zone is challenging. As shown in **Figure 3A**, APTw values remained significantly higher in TZ-PCa lesions compared to BPH nodules (T-test, p < 0.001), demonstrating the robustness of APTw in distinguishing malignancy within the central gland. PSA “Gray Zone” (4–10 ng/mL): In patients with indeterminate PSA levels (4–10 ng/mL), standard PSA testing often lacks specificity. Our analysis (**Figure 3B**) revealed that while the diagnostic performance of serum PSA dropped significantly in this subgroup (AUC = 0.75), APTw imaging maintained robust discriminative ability (AUC = 0.80), effectively stratifying patients who might otherwise undergo unnecessary biopsies.

### 3.5 Correlation between APTw values and Gleason score

**Figure 4:**
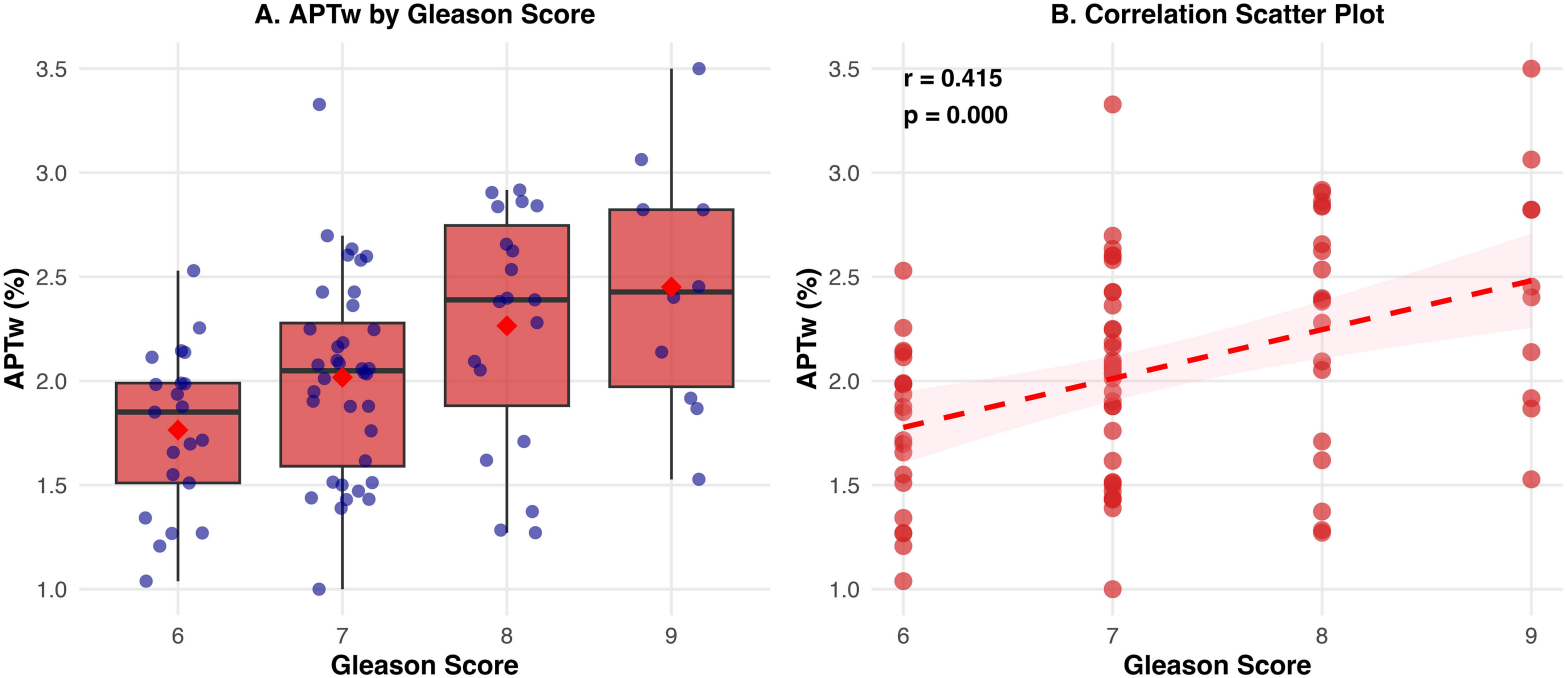
Correlation between APTw values and tumor aggressiveness (Gleason Score). **(A)** Box plot showing a stepwise increase in APTw signal intensity from low-grade to high-grade tumors. **(B)** Scatter plot with linear regression visually corroborating the significant positive correlation between APTw values and Gleason scores (r = 0.415, p < 0.001).

A significant positive correlation was observed between APTmean values and Gleason scores (GS) in the PCa cohort (Spearman’s ρ = 0.407, p < 0.001; Pearson’s r = 0.415, p < 0.001). The distribution of APTw values across different Gleason scores is presented in **Figure 4A**, showing a stepwise trend of increasing APTw signal intensity from low-grade to high-grade tumors. The scatter plot with linear regression (**Figure 4B**) visually corroborates this positive correlation, suggesting that APTw values reflect tumor biological aggressiveness.

### 3.6 Multivariate logistic regression analysis

Multivariate logistic regression was conducted to identify independent diagnostic predictors. The analysis confirmed that APTw, PI-RADS v2.1, and PSA were independent predictors of prostate cancer. Specifically, even after adjusting for the strong predictive power of PI-RADS, APTw remained a significant independent factor (OR > 1, p < 0.05), further supporting its complementary role in the multi-parametric diagnostic model.

### 3.7 Differentiation between high-risk and low- and intermediate-risk prostate cancer

**Figure 5:**
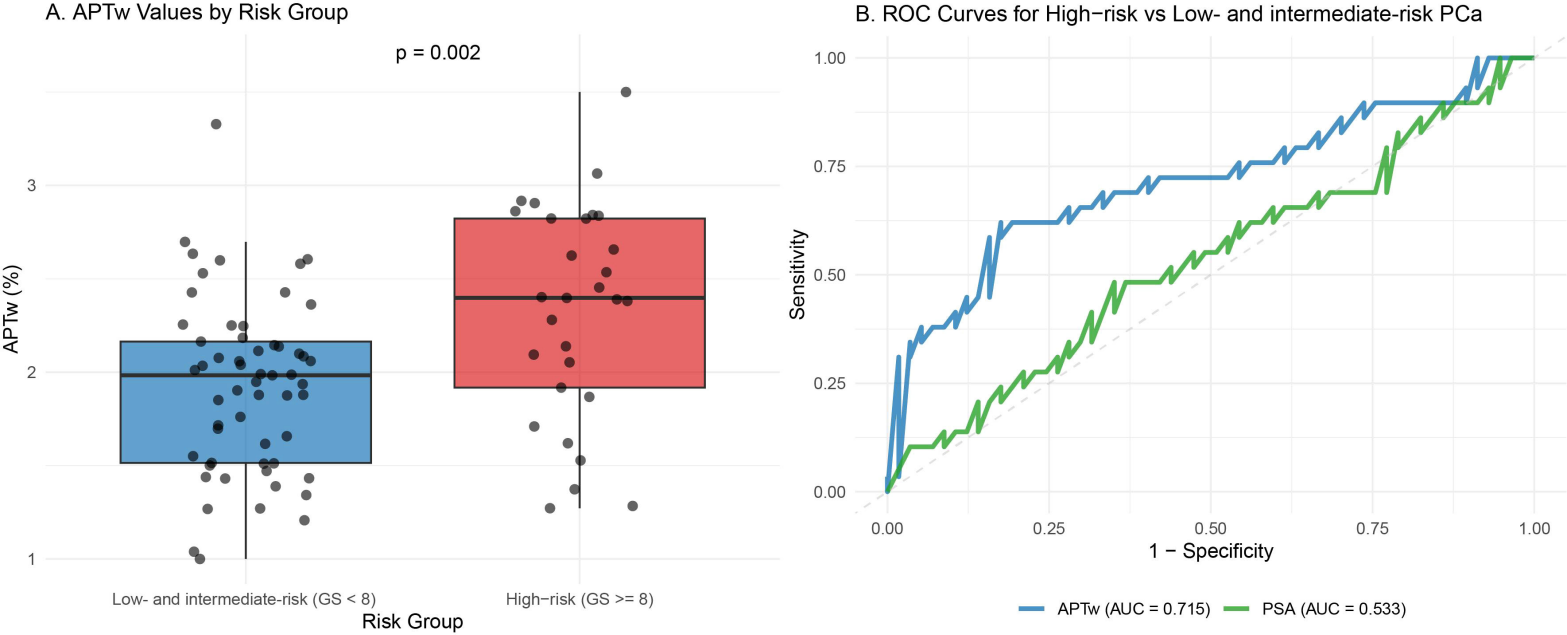
Comparative analysis of APTw imaging in prostate cancer risk stratification. **(A)** Box plot comparing APTw values between low- and intermediate-risk (GS < 8) and high-risk (GS ≥ 8) prostate cancer groups. High-risk tumors demonstrate significantly elevated APTw values (p = 0.002). **(B)** ROC curves showing that APTw (AUC = 0.715) effectively discriminates high-risk disease, whereas serum PSA (AUC = 0.533) shows limited discriminative value.

For distinguishing high-risk from low- and intermediate-risk prostate cancer, APTw imaging demonstrated moderate diagnostic utility with an AUC of 0.715 (95% CI: 0.589-0.841), achieving 62.1% sensitivity and 82.5% specificity at a 2.27% cutoff (**Figure 5A**). The high-risk group registered significantly higher APTw values (2.33% ± 0.58% vs. 1.92% ± 0.46%, p < 0.001), whereas neither serum PSA levels (14.1 ± 7.4 vs. 15.0 ± 7.4 ng/mL, p = 0.62) nor patient age (73.9 ± 3.7 vs. 73.0 ± 5.1 years, p = 0.36) differed significantly. In comparison, PSA exhibited limited discriminative capability (AUC = 0.533, 95% CI 0.400-0.666), similar to the level of random chance (**Figure 5B**). These results indicate APTw’s potential added value in identifying high-risk disease to guide risk-adapted clinical management.

### 3.8 Representative imaging cases

**Figure 6:**
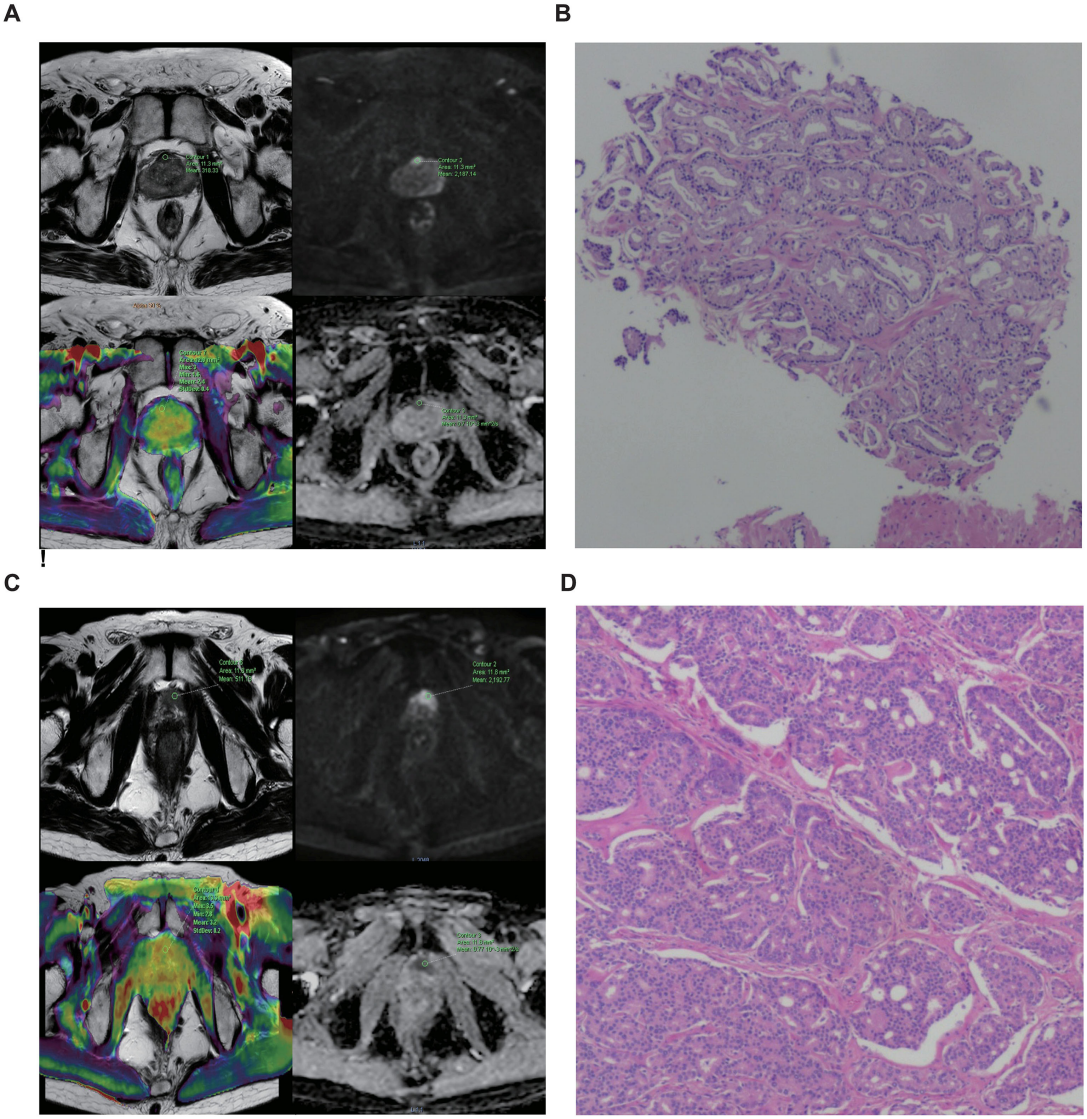
Representative imaging cases of prostate cancer with different Gleason scores. **(A, B)** GS 7 case (65-year-old): **(A)** MRI image. **(B)** Represent the H&E image for diagnosis. **(C, D)** GS 8 case (74-year-old): **(C)** MRI image. **(D)** Represent the H&E image for diagnosis. The higher APTw values in the GS 8 case visually illustrate the positive correlation between APTw and tumor aggressiveness.

**Figure 6** presents two representative cases. Case 1 (GS 3 + 4 = 7, 65-year-old male) shows a lesion with restricted diffusion (hypointense on T2WI, hyperintense on DWI, hypointense on ADC) and a mean APTw value of 2.4% (**Figures 6A, B**). Case 2 (GS 4 + 4 = 8, 74-year-old male) demonstrates more pronounced imaging abnormalities and a higher mean APTw value of 3.2% (**Figures 6C, D**), visually corroborating the quantitative correlation between APTw and tumor aggressiveness.

## 4 Discussion

The present study demonstrates that APTw imaging, as a non-invasive molecular MRI technique, provides significant diagnostic value in the evaluation of prostate cancer. Our findings indicate that APTw values are significantly elevated in PCa compared to BPH and correlate positively with the Gleason Score. Most importantly, this study highlights the incremental value of APTw imaging over standard multi-parametric MRI assessments: the combined model of APTw and PI-RADS v2.1 achieved the highest diagnostic accuracy, and APTw imaging maintained robust performance in clinically challenging scenarios, such as Transition Zone lesions and the PSA “gray zone”.

The observed elevation of APTw signals in PCa compared to BPH (2.1% vs. 1.3%) can be attributed to the fundamental cytological differences between the two conditions. Malignant prostate tissue is characterized by uncontrolled cell proliferation and increased synthesis of cytosolic proteins, which provide abundant exchangeable amide protons (11, 12). In contrast, BPH nodules, despite being hyperplastic, often contain significant stromal components and cystic changes with lower protein concentration relative to water content, resulting in comparatively lower APTw magnetization transfer effects (21). This biological basis supports APTw as a functional surrogate for tumor cellularity and aggressiveness. However, it is important to acknowledge that the APTw signal, calculated via traditional asymmetry analysis, is a mixed contrast rather than an absolute quantification of mobile protein concentration. The measured signal intensity is inherently influenced by several confounding physiochemical factors, including tissue pH, concurrent semi-solid magnetization transfer (MT) effects, and water T1 relaxation properties (26, 27). For instance, while high-grade tumors exhibit elevated protein content, concurrent changes in the acidic microenvironment (pH) can modulate the proton exchange rate, thereby affecting the final APTw contrast. Therefore, future studies incorporating advanced multipool CEST modeling may be required to decouple these effects and further refine the exact molecular interpretation of prostate lesions (28).

Regarding diagnostic performance, our study confirms that APTw imaging (AUC = 0.840) serves as a robust biomarker. However, a critical question addressed in this study is whether APTw adds value to the current clinical standard, PI-RADS v2.1. While PI-RADS v2.1 alone demonstrated excellent performance (AUC = 0.875), the integration of APTw into a combined model significantly improved diagnostic efficacy (AUC = 0.915, p < 0.05). This suggests that APTw provides complementary metabolic information that anatomical (T2WI) and diffusion (DWI) sequences may miss. For instance, in cases where inflammation or stromal hyperplasia mimics cancer on DWI (false positives), the relatively lower protein content of benign tissues may result in lower APTw values, helping to rule out malignancy.

A key strength of this study is the subgroup analysis addressing specific clinical dilemmas. Differentiating PCa from BPH in the Transition Zone (TZ) is notoriously difficult due to the heterogeneous background of stromal hyperplasia (8). Our results showed that APTw values significantly differentiated TZ-PCa from BPH nodules (p < 0.001), suggesting its potential utility in the central gland. Furthermore, in patients with PSA levels in the “gray zone” (4–10 ng/mL), where the specificity of serum PSA is notoriously poor (AUC = 0.75 in our cohort), APTw imaging maintained high diagnostic accuracy (AUC = 0.80). This finding implies that incorporating APTw into the diagnostic workflow for gray-zone patients could potentially reduce unnecessary biopsies.

Risk stratification is crucial for treatment planning. Our study found a moderate positive correlation between APTw values and Gleason Scores (r = 0.415), consistent with previous findings in gliomas and prostate cancer (22, 29). When distinguishing high-risk (GS ≥ 8) from low- and intermediate-risk PCa, APTw imaging achieved an AUC of 0.715, whereas serum PSA failed to discriminate between the groups (AUC = 0.533). This indicates that while PSA reflects prostate volume and inflammation, APTw is more directly linked to the biological aggressiveness of the tumor.

We acknowledge several limitations in our study. First, as a single-center retrospective study, potential selection bias is unavoidable, and the patient cohort may not fully represent the general screening population. Second, although our overall sample size was sufficient for primary analysis, the number of patients in the specific “gray zone” subgroup and the subgroup of high-risk (GS ≥ 8) cancers were relatively modest, potentially limiting the statistical power for stratified analyses. Third, and most importantly, we lacked an external validation cohort. While our internal cross-validation and comparison with PI-RADS provided promising results, further multi-center prospective studies involving larger and more diverse cohorts using scanners from different vendors are essential to externally validate these early thresholds and establish the reproducibility of the proposed APTw cut-off values before widespread clinical adoption.

In conclusion, APTw imaging is a valuable addition to the multi-parametric MRI of the prostate. It not only differentiates PCa from BPH with high accuracy but also provides incremental value to PI-RADS v2.1 and demonstrates robust performance in challenging clinical subgroups.

## Data Availability

The original contributions presented in the study are included in the article/**Supplementary Material**. Further inquiries can be directed to the corresponding author.

## References

[1] Bergengren O Bray F Brierley J Dillner J Eklund M Engholm G . 2022 update on prostate cancer epidemiology and risk factors-A systematic review. Eur Urol. (2023) 84:191–206. doi: 10.1016/j.eururo.2023.04.021, PMID: 37202314 PMC10851915

[2] Schaeffer EM Srinivas S Adra N An Y Barocas D Bitting R . NCCN guidelines^®^ Insights: prostate cancer, version 1.2023. J Natl Compr Canc Netw. (2022) 20:1288–98. 10.6004/jnccn.2022.0063 36509074

[3] Hurwitz LM Pinsky PF Trabert B . Recommended definitions of aggressive prostate cancer for etiologic epidemiologic research. J Natl Cancer Inst. (2021) 113:727–34. doi: 10.1093/jnci/djaa154, PMID: 33010161 PMC8248961

[4] Godtman RA Kollberg KS Pihl C-G Månsson M Hugosson J . The association between age, prostate cancer risk, and higher gleason score in a long-term screening program: results from the göteborg-1 prostate cancer screening trial. Eur Urol. (2022) 82:311–7. doi: 10.1016/j.eururo.2022.01.018, PMID: 35120773

[5] Bernhardt M Sakr M Albadry R Gordetsky J Wu H Kaushal S . Unexpectedly high variability in determining tumour extent in prostatic biopsies: implications for active surveillance. Histopathology. (2025) 86:627–39. doi: 10.1111/his.15372, PMID: 39610035 PMC11791730

[6] Niu J Xu H Wu Y Cheng X Cao Q Wang Y . Preoperative magnetic resonance imaging pelvic and prostatic parameters predict long-term urinary continence after Retzius-sparing robot-assisted radical prostatectomy. Sci Rep. (2025) 15:13543. doi: 10.1038/s41598-025-91837-9, PMID: 40253503 PMC12009336

[7] Bekou E Seimenis I Tsochatzis A Tziagkana K Kelekis N Deftereos S . The role of radiomic analysis and different machine learning models in prostate cancer diagnosis. J Imaging. (2025) 11:250. doi: 10.3390/jimaging11080250, PMID: 40863460 PMC12387180

[8] Nam R Wallis C Saskin R Herschorn S Kodama R Lee Y . Prostate MRI versus PSA screening for prostate cancer detection (the MVP Study): a randomised clinical trial. BMJ Open. (2022) 12:e059482. doi: 10.1136/bmjopen-2021-059482, PMID: 36351725 PMC9644313

[9] Koike H Togao O Hiwatashi A Yamashita K Kikuchi K Momosaka D . Amide proton transfer-chemical exchange saturation transfer imaging of intracranial brain tumors and tumor-like lesions: our experience and a review. Diagnostics. (2023) 13:881. doi: 10.3390/diagnostics13050914, PMID: 36900058 PMC10000843

[10] Meng N Wang X Sun J Liu M Zheng L Wang X . Application of the amide proton transfer-weighted imaging and diffusion kurtosis imaging in the study of cervical cancer. Eur Radiol. (2020) 30:5758–67. doi: 10.1007/s00330-020-06884-9, PMID: 32424593

[11] Zhou J Payen J-F Wilson DA Traystman RJ van Zijl PCM . Using the amide proton signals of intracellular proteins and peptides to detect pH effects in MRI. Nat Med. (2003) 9:1085–90. doi: 10.1038/nm907, PMID: 12872167

[12] Park JE Kim HS Park KJ Kim SJ Kim JH Smith SA . Pre- and posttreatment glioma: comparison of amide proton transfer imaging with MR spectroscopy for biomarkers of tumor proliferation. Radiology. (2016) 278:514–23. doi: 10.1148/radiol.2015142979, PMID: 26491847

[13] Su C Liu C Zhao L Jiang C Cai J Wang K . Amide proton transfer imaging allows detection of glioma grades and tumor proliferation: comparison with ki-67 expression and proton MR spectroscopy imaging. AJNR Am J Neuroradiol. (2017) 38:1702–9. doi: 10.3174/ajnr.A5301, PMID: 28729292 PMC7963688

[14] Saffarian S Cai Z Lam J Oo HZ Somasekharan S . Elevated NPM1 and FBL expression correlates with prostate cancer aggressiveness and progression. J Pathol. (2025) 267:56–68. doi: 10.1002/path.6447, PMID: 40705480 PMC12337817

[15] Alhamdani Z Poppenbeek S Bolton D Wong L-M Sethi K . Do alpha blockers reduce the risk of urinary retention post-transperineal prostate biopsy? A systematic narrative review. World J Urol. (2024) 42:332. doi: 10.1007/s00345-024-05001-5, PMID: 38758413 PMC11101363

[16] Mian BM Ayyash OM Halpern DM Hsu C-H Li H Batura D . Complications following transrectal and transperineal prostate biopsy: results of the proBE-PC randomized clinical trial. J Urol. (2024) 211:205–13. doi: 10.1097/JU.0000000000003788, PMID: 37976319

[17] Ding C-KC Verduzco R Mendez A Kao CS Sharma R Gupta N . Classification of prostatic adenocarcinoma as favourable/unfavourable histology has high interobserver agreement in prostate needle core biopsies. Histopathology. (2025) 87:717–25. doi: 10.1111/his.15525, PMID: 40762236 PMC12522031

[18] Marino F Maida FD Licari LC Cangemi A Urzi D Cimino S . Robot-assisted radical prostatectomy performed with the novel hugo™ RAS system: A systematic review and pooled analysis of surgical, oncological, and functional outcomes. J Clin Med. (2024) 13:1621. doi: 10.3390/jcm13092551, PMID: 38731080 PMC11084580

[19] Hafiani H Hafiani I Hafiani M . Sarcomatoid prostate carcinoma: a case report. BMC Urol. (2025) 25:104. doi: 10.1186/s12894-025-01790-y, PMID: 40281516 PMC12023537

[20] Zhou W Yang Z Li J Chen B Wu C Lu H . A visualized machine learning model using noninvasive parameters to differentiate men with and without prostatic carcinoma before biopsy. Sci Rep. (2025) 15:27357. doi: 10.1038/s41598-025-12765-2, PMID: 40717135 PMC12301460

[21] Jia G Abaza R Williams JD Zynger DL Zhou J Shah ZK . Amide proton transfer MR imaging of prostate cancer: a preliminary study. J Magn Reson Imaging. (2011) 33:647–54. doi: 10.1002/jmri.22480, PMID: 21563248 PMC4287206

[22] Takayama Y Nishie A Sugimoto M Togao O Asayama Y Ishigami K . Amide proton transfer (APT) magnetic resonance imaging of prostate cancer: comparison with Gleason scores. Magn Reson Mater Phy. (2016) 29:671–9. doi: 10.1007/s10334-016-0537-4, PMID: 26965511

[23] van Leenders GJLH van der Kwast TH Grignon DJ Evans AJ Kristiansen G Kweldam CF . The 2019 international society of urological pathology (ISUP) consensus conference on grading of prostatic carcinoma. Am J Surg Pathol. (2020) 44:e87–99. doi: 10.1097/PAS.0000000000001497, PMID: 32459716 PMC7382533

[24] Bostwick DG Foster CS . Predictive factors in prostate cancer: current concepts from the 1999 College of American Pathologists Conference on Solid Tumor Prognostic Factors and the 1999 World Health Organization Second International Consultation on Prostate Cancer. Semin Urol Oncol. (1999) 17:222–72. 10632123

[25] R Core Team . R: A language and environment for statistical computing. Vienna, Austria: R Foundation for Statistical Computing (2022). Available online at: https://www.R-project.org/ (Accessed October 1, 2025).

[26] van Zijl PC Yadav NN . Chemical exchange saturation transfer (CEST): what is in a name and what isn’t? Magn Reson Med. (2011) 65:927–48. doi: 10.1002/mrm.22761, PMID: 21337419 PMC3148076

[27] Wu B Warnock G Zaiss M Lin C Chen M Zhou Z . An overview of CEST MRI for non-MR physicists. EJNMMI Phys. (2016) 3:19. doi: 10.1186/s40658-016-0155-2, PMID: 27562024 PMC4999387

[28] Zaiss M Windschuh J Paech D Meissner JE Burth S Schmitt B . Relaxation-compensated CEST-MRI of the human brain at 7T: Unbiased insight into NOE and amide signal changes in human glioblastoma. Neuroimage. (2015) 112:180–8. doi: 10.1016/j.neuroimage.2015.02.040, PMID: 25727379

[29] Togao O Yoshiura T Keupp J Hiwatashi A Yamashita K Kikuchi K . Amide proton transfer imaging of adult diffuse gliomas: correlation with histopathological grades. Neuro Oncol. (2014) 16:441–8. doi: 10.1093/neuonc/not158, PMID: 24305718 PMC3922507

